# Synthesis and structure of 1-(2-bromo­phen­yl)-2-chloro-3-(2-chloracet­yl)-1*H*-indole

**DOI:** 10.1107/S2056989016018880

**Published:** 2016-11-30

**Authors:** Ting-ting Zhang, Bing Wang, Qing Lu, Jun-fang Zhao, Hong Lei, Qi Fang

**Affiliations:** a School of Chemistry and Chemical Engineering, Shandong University, Jinan 250100, People’s Republic of China; b State Key Laboratory of Crystal Materials, Shandong University, Jinan 250100, People’s Republic of China; c Technical Institute of Physics and Chemistry, Chinese Academy of Science, Beijing 100190, People’s Republic of China

**Keywords:** synthesis, crystal structure, indole derivative, hydrogen bonding, centrosymmetric dimer

## Abstract

In the title indole derivative, C_16_H_10_BrCl_2_NO, the dihedral angle between the mean plane of the indole ring system and the mean plane of the disordered 2-bromo­phenyl ring is 77.6 (1)°. The non-H atoms of the chloracetyl group are essentially coplanar with the indole core. In the crystal, pairs of mol­ecules are face-to-face embraced *via* two weak C—H⋯O hydrogen bonds, forming inversion dimers with an inter­planar distance between two parallel indole planes of 3.360 (3) Å. These dimers are connected by head-to-head Cl⋯Cl inter­molecular contacts to build a two-dimensional mol­ecular sheet parallel to (101). Neighbouring mol­ecular sheets are stacked together to construct the three-dimensional structure by further short Cl⋯Cl inter­molecular contacts. The atoms of the bromo­phenyl group were refined as disordered over two sets of sites with refined occupancies of 0.61 (2) and 0.39 (2).

## Abstract 2

In this indole derivative, the dihedral angle between the mean plane of the indole ring system and the mean plane of the benzene ring of the 2-bromo­phenyl group is 77.6 (1)°. In the crystal, pairs of mol­ecules are face-to-face embraced *via* two weak C—H⋯O hydrogen bonds, forming inversion dimers which are connected by head-to-head Cl⋯Cl inter­molecular contacts to build a mol­ecular sheet parallel to (101). Neighbouring sheets are stacked together by further short Cl⋯Cl inter­molecular contacts to construct the three-dimensional structure.

## Chemical context   

Indole derivatives occur in many natural products and they have been widely used as inter­mediates in the pharmaceutical industry (Chaskar *et al.*, 2010 ▸). Indolyl is the base skeleton of tryptophan, which is one of the essential amino acids of human beings. In addition, indole derivatives such as indole-3-acetic acid (Won *et al.*, 2011 ▸), serotonin (Batsikadze *et al.*, 2013 ▸) and melatonin (Diss *et al.*, 2013 ▸) act as hormones existing in different kinds of plants and animals. Some indole derivatives show anti­carcinogenic, hypotensive and anti­neoplastic activities (Zhang *et al.*, 2015 ▸). The indole skeleton can be found in many bioactive drugs, such as ajmalicine (Du *et al.*, 2014 ▸), vinblastine (Ishikawa *et al.*, 2008 ▸) and reserpine (Chen & Huang, 2005 ▸).
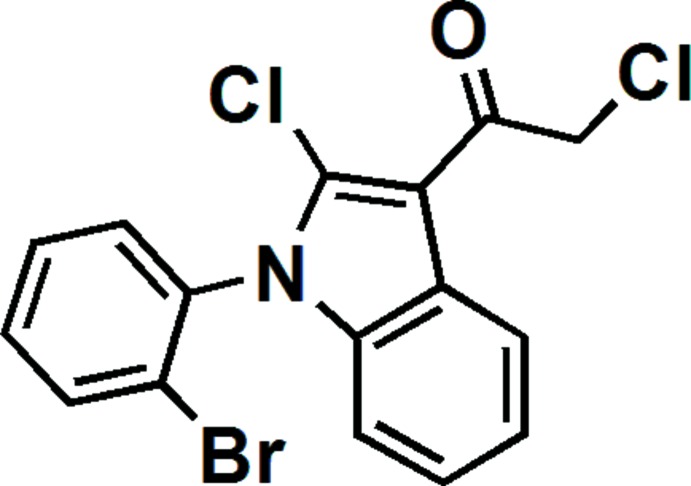


To date, a collection of more than six thousand structures with the 1*H*-indole substructure have been recorded in the Cambridge Structural Database (CSD; Groom *et al.*, 2016 ▸), which includes a subset of more than one hundred structures containing the 1-phenyl-1*H*-indole substructure, including as 1-phenyl-indolin-2-one and several derivatives, reported by our group (Wang *et al.*, 2015 ▸). However, the structures of halogen-substituted 1-phenyl-1*H*-indole derivatives, such as 2-iodo-1-phenyl-1*H*-indole (Messaoud *et al.*, 2015 ▸) are very limited. Recently, we unexpectedly synthesized the new title compound, 1-(2-bromo­phen­yl)-2-chloro-3-(2-chloracet­yl)-1*H*-indole. Herein we report its synthesis and crystal structure.

## Structural commentary   

**Figure 1:**
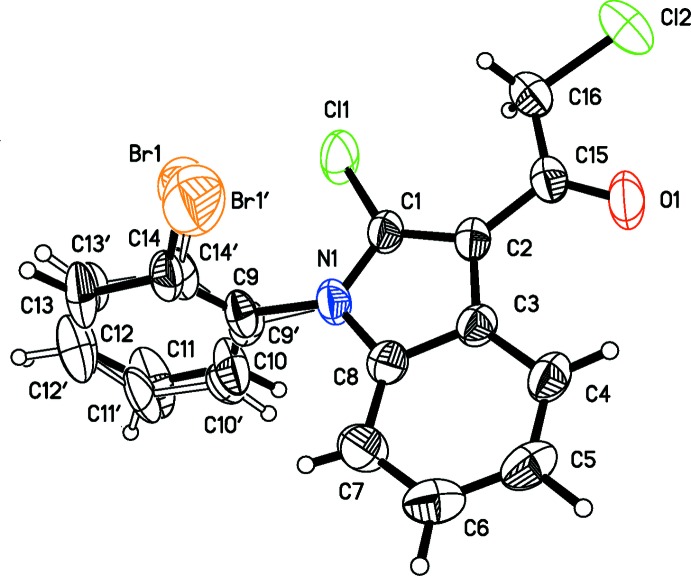
The mol­ecular structure of the title compound. Displacement ellipsoids are drawn at the 50% probability level. The minor component of disorder is shown with open bonds.

As shown in Fig. 1 ▸, the mol­ecule consists basically of two planes, the indole unit and the phenyl ring. Nine non-H atoms (N1/C1–C8) are essentially planar and their mean plane defines the indole plane. Five more non-H atoms are approximately co-planar with the indole core with deviations of −0.050 (2) Å for C15, 0.067 (1) Å for Cl1, 0.032 (1) Å for O1, −0.190 (2) Å for C16, and −0.355 (1) Å for Cl2. The C4—H4⋯O1 short intermolecular contact (see Table 1 ▸) plays an important role in keeping the four non-H atoms of chloracetyl co-planar with the indole plane. The mean plane of the 2-bromophenyl ring (defined as the mean plane of the six C atoms of the major component and six C atoms of the minor component of the disordered benzene ring of the 2-bromo­phenyl group) subtends a dihedral angle of 77.6 (1)° to the indole plane.

The deviation of atom N1 from the C1,C8,C9 triangle is very small [0.005 (2) Å], indicating *sp* ^2^ hybridization of this atom. The five-membered ring of the indole core shows similar bond-length characteristics to those of the reference structure 2-iodo-1-phenyl-1*H*-indole (Messaoud *et al.*, 2015 ▸). The C1=C2 bond [1.374 (2) Å] is slightly longer than a double bond and longer than that of the reference structure. This is because of certain C1 C2 C15 π-conjugation of the three atoms, revealed by the shorter single bond C2—C15 [1.463 (2) Å]. The C1—N1 bond shows strong double-bond character with a length of 1.365 (2) Å while C8—N1 [1.3939 (19) Å] is shorter than a single C—N bond. Both the C1—N1 and the C8—N1 bond lengths are shorter than those of the reference structure.

## Supra­molecular features   

**Figure 2:**
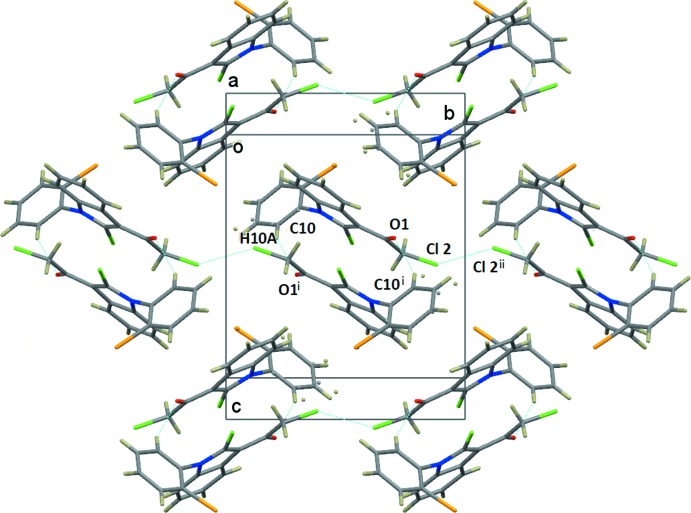
A view along the a* direction, showing the C10—H10*A*⋯O1^i^ hydrogen bond in a dimer and the Cl2⋯Cl2^ii^ short contact forming chains along the *b*-axis direction. [Symmetry codes: (i) −*x* + 1, −*y* + 1, −*z* + 1; (ii) −*x* + 1, −*y* + 2, −*z* + 1.]

**Figure 3:**
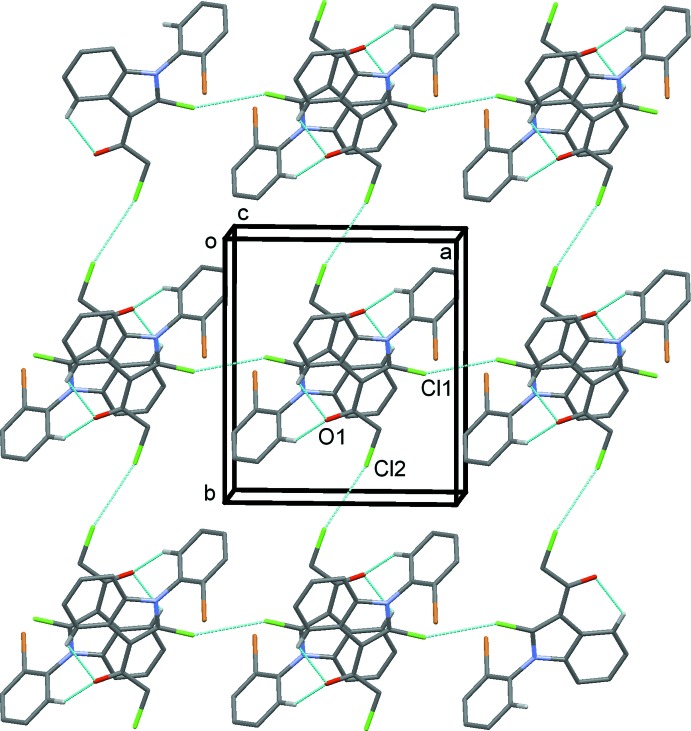
A view along the *c*-axis direction, showing the C—H⋯O hydrogen bonds (see Table 1 ▸) and Cl⋯Cl contacts as dashed lines. Only H atoms H4 and H10*A* have been included. The C atoms of the minor component of the disordered benzene ring have been omitted.

In the crystal, pairs of mol­ecules are connected by two C—H⋯O hydrogen bonds (see Table 1 ▸) and are face-to-face embraced to form an inversion dimer, with the inter­planar spacing between the two parallel indole planes being 3.360 (3) Å (see Fig. 2 ▸). Neighbouring dimers are further connected by a type of head-to-head Cl2⋯Cl2 (−*x* + 1, −*y* + 2, −*z* + 1) short contacts of 3.3759 (8) Å, forming chains along the *b*-axis direction. As shown in Fig. 3 ▸, the chains are linked by further side-by-side Cl1⋯Cl1(−*x* + 2, −*y* + 1, −*z* + 1) [3.3430 (7) Å] short contacts, forming sheets parallel to the *ab* plane.

The inter­molecular inter­actions can be scaled by the electronic transfer integrals (*t*) between two neighbouring mol­ecules and can be calculated according to Deng & Goddard, 2004 ▸) as *t* = (*E*_HOMO_ − *E*_HOMO-1_)/2 where *E*_HOMO_ and *E*_HOMO-1_ are the energy levels of the HOMO (highest occupied mol­ecular orbital) and the HOMO-1 orbital of a two-mol­ecule pair, respectively. The calculation was carried out by DFT methods at the level of b31yp/6-311g(d) using the *GAUSSIAN03* program (Frisch *et al.*, 2003 ▸). The *t* values for the face-to-face mol­ecular pair (the dimer), the Cl2⋯Cl2 head-to-head pair, and the Cl1⋯Cl1 side-by-side pair were calculated to be 0.051, 0.00053, 0.00076 eV, respectively. This indicates that the inter­molecular inter­actions of the dimer are the strongest.

**Figure 4:**
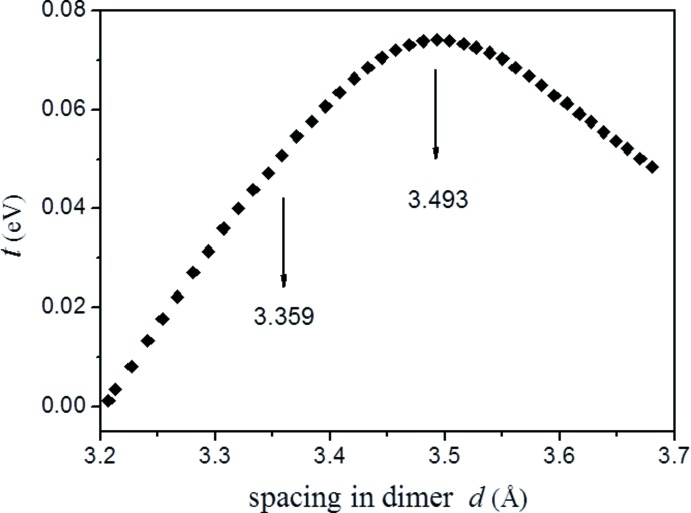
The evolution of the calculated electronic transfer integrals (*t*) as a function of spacing between the two mol­ecules of the face-to-face dimer. The optimized spacing at the peak *t*-curve and the spacing in the X-ray structure are indicated.

Fig. 4 ▸ shows the calculated electronic transfer integrals (*t*) of an isolated face-to-face dimer *versus* the spacing between the two indole planes of the dimer. When varying the spacing, the mol­ecular configuration is fixed to the X-ray mol­ecular structure that resulted from a non-disorder refinement. The spacing (3.493 Å) at the peak of the *t-*curve is slightly larger than the spacing [3.359 (3) Å] in the X-ray structure, indicating a shrinking of the spacing of the dimer when the crystal packing is concerned.

## Database survey   

A search of the Cambridge Structural Database (WebCSD, last update 2016-10-26) for the substructure of the non-H 1*H*-indole skeleton gave 6467 hits. There are 151 structures which contain the 1-phenyl-1*H*-indole substructure. The only structure of the 2-halogen-1-phenyl-1*H*-indole type is 2-iodo-1-phenyl-1*H*-indole (Messaoud *et al.*, 2015 ▸) and no structure for the title compound. There are no records of this compound in the SciFinder Database.

## Synthesis and crystallization   

**Figure 5:**
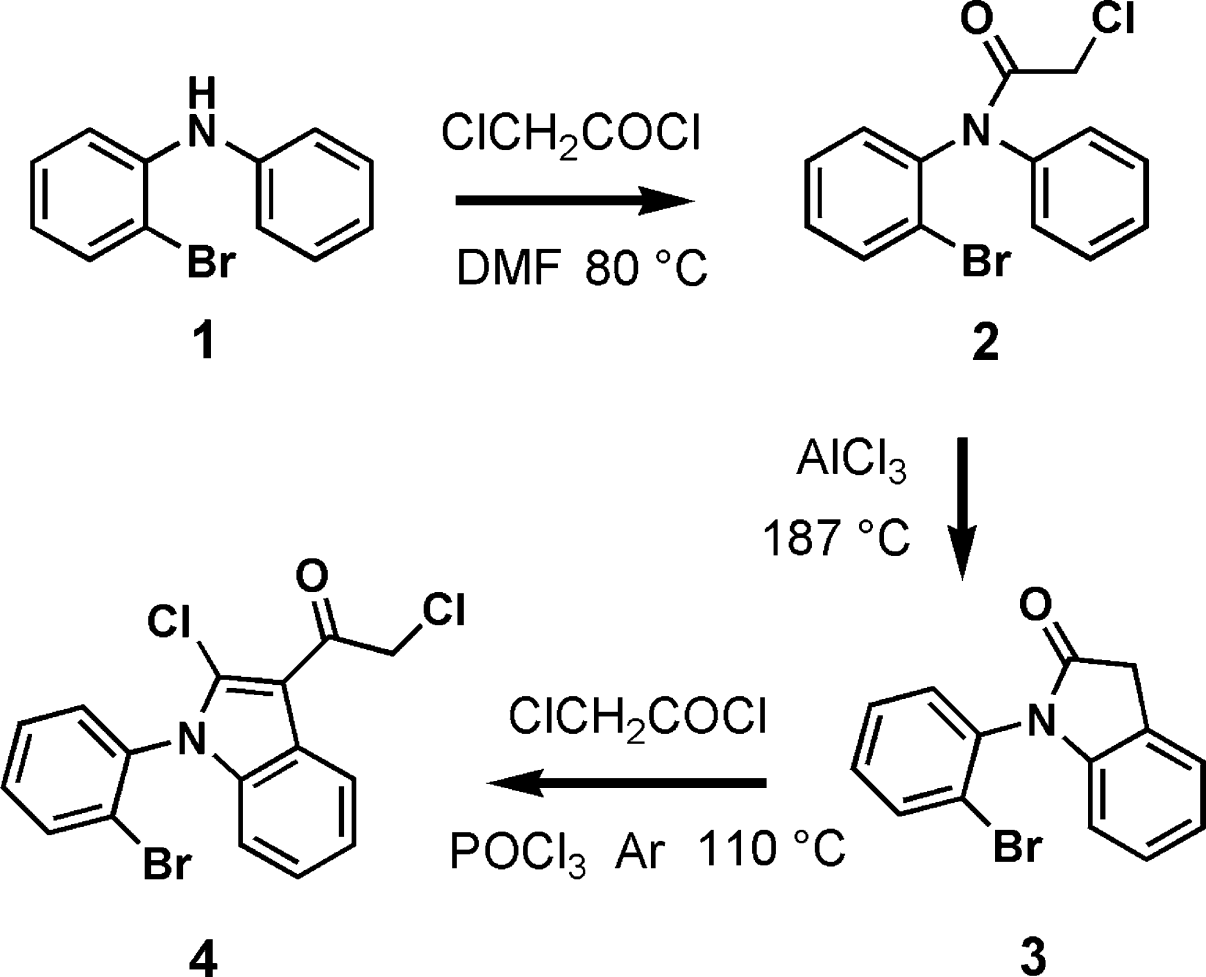
Reaction scheme.

The title compound was synthesized in three steps (see Fig. 5 ▸). Firstly, compound **2** was synthesized by acyl­ation of compound **1** with chloracetyl chloride in *N*, *N*-di­methyl­acetamide (DMF). Compound **1** (6.58 g, 26.5 mmol), chloracetyl chloride (3.2 mL, 40 mmol), and DMF solvent (2 mL) were added into a 250 mL flask and the mixture was stirred at 353 K for 2 h. Then 200 mL water was added into the mixture and it was kept stirring for 0.5 h. The colorless products (13.9 g) were compound **2** together with some unreacted chloracetyl chloride.

Secondly, a Friedel–Crafts reaction of compound **2**, under the catalysis of anhydrous AlCl_3_, resulted in the ring-closure compound **3**. To a 250 mL flask, compound **2** (8.22 g, 25.4 mmol) and anhydrous AlCl_3_ (10.15 g, 76.1 mmol) were added and stirred mechanically for 15 minutes at 460 K. The mixture was poured into 200 mL water and extracted with CH_2_Cl_2_. The crude product was purified by silica gel column chromatography with ethyl acetate and petroleum ether (*v*/*v* = 1:10) as eluent. Compound **3** was obtained together with some residual chloracetyl chloride (3.50 g in all).

**Figure 6:**
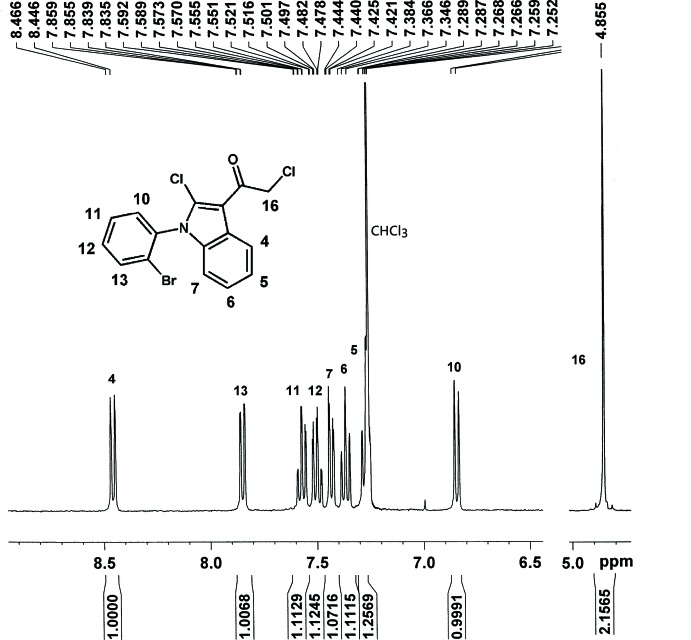
The ^1^H NMR spectra of the title compound.

Finally, the title compound **4** was obtained as a by-product of trimerization of compound **3** in the presence of POCl_3_ and chloracetyl chloride. As shown in Fig. 5 ▸, the Cl atom bonded to the indole core should come from POCl_3_, which is supported by our other experiment. Compound **3** (0.92 g, 3.2 mmol) and 6 mL POCl_3_ were added into a 100 mL Schlenk tube and the mixture was stirred at 383 K in an argon atmosphere for 9 h. After cooling, the mixture was poured into 500 mL ice–water and stirred intensely until a black solid appeared. The solid was dissolved in CH_2_Cl_2_, washed with water and dried with MgSO_4_. The solvent was removed and the crude solid was initially separated by silica gel column chromatography with ethyl acetate and petroleum ether (*v*/*v* = 1:100) as eluent to obtain a mixture, which consists of the compound of trimerization (will be reported elsewhere) and the title compound **4**. The colorless crystals of compound **4** (0.0093 g, m. p. 456–458 K), which were suitable for X-ray structure determination, were obtained by a silica gel column chromatography of the above mixture with *n*-hexane as eluent, following a quick evaporation of the *n*-hexane solution overnight. ^1^H NMR (400 MHz, CDCl_3_) δ 8.46 (*d*, *J* = 8.0 Hz, 1H), 7.85 (*d*, *J* = 8.0 Hz, 1H), 7.57 (*t*, *J* = 15.2 Hz, 1H), 7.50 (*t*, *J* = 15.2 Hz, 1H), 7.43 (*d*, *J* = 7.6 Hz, 1H), 7.37 (*t*, *J* = 15.2 Hz, 1H), 7.27(*t*, *J* = 16.0 Hz, 1H), 6.85 (*d*, *J* = 8.0 Hz, 1H), 4.86 (*s*, 1H). As shown in Fig. 6 ▸, the ^1^H NMR signals of all protons of the title compound are well separated and well characterized.

## Refinement   

Crystal data, data collection and structure refinement details are summarized in Table 2 ▸. H atoms of the disordered benzene ring were placed at calculated positions and refined using a riding-model approximation with C—H = 0.93 Å and *U*_iso_ = 1.2*U*_eq_(C). All other H atoms were located in difference maps and freely refined, leading to C—H distances from 0.85 (2) to 1.08 (2) Å. The 2-bromo­phenyl group was refined as disordered over two sets of sites, which gave better results (*R*_1_ = 0.032, Δρ_max_= 0.27). By comparison, the results of the non-disordered treatment were relatively poor (*R*_1_ = 0.043, Δρ_max_= 0.93). However, the non-disordered mol­ecular geometry was used for DFT calculation in this work.

## Supplementary Material

Crystal structure: contains datablock(s) I. DOI: 10.1107/S2056989016018880/lh5827sup1.cif

Structure factors: contains datablock(s) I. DOI: 10.1107/S2056989016018880/lh5827Isup2.hkl

Supporting information file. DOI: 10.1107/S2056989016018880/lh5827Isup3.cml

CCDC reference: 1519194

Additional supporting information: crystallographic information; 3D view; checkCIF report

## Figures and Tables

**Table 1:** Hydrogen-bond geometry (Å, °)

*D*—H⋯*A*	*D*—H	H⋯*A*	*D*⋯*A*	*D*—H⋯*A*
C4—H4⋯O1	0.92 (2)	2.502 (19)	3.053 (2)	118.5 (15)
C10—H10*A*⋯O1^i^	0.93	2.45	3.302 (7)	152

Symmetry code: (i) 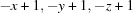.

**Table 2:** Experimental details

Crystal data	
Chemical formula	C_16_H_10_BrCl_2_NO
*M*_r_	383.06
Crystal system, space group	Monoclinic, *P*2_1_/*n*
Temperature (K)	297
*a*, *b*, *c* (Å)	10.2155 (10), 11.3645 (11), 13.5252 (13)
β (°)	101.141 (2)
*V* (Å^3^)	1540.6 (3)
*Z*	4
Radiation type	Mo *K*α
μ (mm^−1^)	3.01
Crystal size (mm)	0.48 × 0.41 × 0.38

Data collection	
Diffractometer	Bruker APEXII CCD
Absorption correction	Multi-scan (*APEX2*; Bruker, 2005 ▸)
*T*_min_, *T*_max_	0.324, 0.398
No. of measured, independent and observed [*I* > 2σ(*I*)] reflections	19200, 4126, 2654
*R*_int_	0.084
(sin θ/λ)_max_ (Å^−1^)	0.689

Refinement	
*R*[*F* ^2^ > 2σ(*F* ^2^)], *wR*(*F* ^2^), *S*	0.032, 0.086, 0.94
No. of reflections	4126
No. of parameters	266
No. of restraints	2
H-atom treatment	H atoms treated by a mixture of independent and constrained refinement
Δρ_max_, Δρ_min_ (e Å^−3^)	0.27, −0.37

Computer programs: *APEX2* and *SAINT* (Bruker, 2005 ▸), *SHELXS97*, *SHELXL97* and *SHELXTL* (Sheldrick, 2008 ▸).

## supplementary crystallographic information

### Crystal data

**Table:**

C_16_H_10_BrCl_2_NO	*F*(000) = 760
*M**_r_* = 383.06	*D*_x_ = 1.652 Mg m^−^^3^
Monoclinic, *P*2_1_/*n*	Mo *K*α radiation, λ = 0.71073 Å
Hall symbol: -P 2yn	Cell parameters from 6321 reflections
*a* = 10.2155 (10) Å	θ = 2.3–26.3°
*b* = 11.3645 (11) Å	µ = 3.01 mm^−^^1^
*c* = 13.5252 (13) Å	*T* = 297 K
β = 101.141 (2)°	Parallelpiped, colourless
*V* = 1540.6 (3) Å^3^	0.48 × 0.41 × 0.38 mm
*Z* = 4	

### Data collection

**Table:**

Bruker APEXII CCD diffractometer	4126 independent reflections
Radiation source: fine-focus sealed tube	2654 reflections with *I* > 2σ(*I*)
Graphite monochromator	*R*_int_ = 0.084
Detector resolution: 8.3 pixels mm^-1^	θ_max_ = 29.3°, θ_min_ = 2.3°
ω scans	*h* = −14→13
Absorption correction: multi-scan (APEX2; Bruker, 2005)	*k* = −15→15
*T*_min_ = 0.324, *T*_max_ = 0.398	*l* = −18→18
19200 measured reflections	

### Refinement

**Table:**

Refinement on *F*^2^	Primary atom site location: structure-invariant direct methods
Least-squares matrix: full	Secondary atom site location: difference Fourier map
*R*[*F*^2^ > 2σ(*F*^2^)] = 0.032	Hydrogen site location: mixed
*wR*(*F*^2^) = 0.086	H atoms treated by a mixture of independent and constrained refinement
*S* = 0.94	*w* = 1/[σ^2^(*F*_o_^2^) + (0.0405*P*)^2^] where *P* = (*F*_o_^2^ + 2*F*_c_^2^)/3
4126 reflections	(Δ/σ)_max_ = 0.002
266 parameters	Δρ_max_ = 0.27 e Å^−^^3^
2 restraints	Δρ_min_ = −0.37 e Å^−^^3^

### Special details

**Table:**

Experimental. Scan width 0.3° ω , Crystal to detector distance 5.964 cm, exposure time 10s, 10 hours for data collection, without scale. The 4 omiga-run take the following theta, initial-omiga, phi values and the following sweep-ranges, respectively -25, -28, 0, 186 (negatively run) -28, 146, 180, 186 (positively run) -33, -28, 90, 186 (negatively run) -33, 127, 270, 220 (positively run)
Geometry. All esds (except the esd in the dihedral angle between two l.s. planes) are estimated using the full covariance matrix. The cell esds are taken into account individually in the estimation of esds in distances, angles and torsion angles; correlations between esds in cell parameters are only used when they are defined by crystal symmetry. An approximate (isotropic) treatment of cell esds is used for estimating esds involving l.s. planes.
Refinement. Refinement of F^2^ against ALL reflections. The weighted R-factor wR and goodness of fit S are based on F^2^, conventional R-factors R are based on F, with F set to zero for negative F^2^. The threshold expression of F^2^ > 2sigma(F^2^) is used only for calculating R-factors(gt) etc. and is not relevant to the choice of reflections for refinement. R-factors based on F^2^ are statistically about twice as large as those based on F, and R- factors based on ALL data will be even larger.

### Fractional atomic coordinates and isotropic or equivalent isotropic displacement parameters (Å^2^)

**Table:**

	*x*	*y*	*z*	*U*_iso_*/*U*_eq_	Occ. (<1)
Br1	0.9017 (3)	0.45726 (16)	0.23888 (15)	0.0680 (7)	0.61 (2)
Br1'	0.8772 (11)	0.4504 (4)	0.2351 (4)	0.1042 (14)	0.39 (2)
Cl1	0.84021 (4)	0.52798 (5)	0.50311 (4)	0.06661 (18)	
Cl2	0.58942 (6)	0.87898 (4)	0.54210 (5)	0.07744 (19)	
O1	0.42385 (12)	0.70363 (11)	0.42434 (10)	0.0616 (4)	
N1	0.68599 (12)	0.39373 (11)	0.37045 (10)	0.0431 (3)	
C1	0.69364 (14)	0.49615 (15)	0.42401 (13)	0.0416 (4)	
C2	0.57445 (15)	0.55577 (13)	0.40546 (13)	0.0391 (4)	
C3	0.48447 (14)	0.48228 (14)	0.33524 (12)	0.0379 (3)	
C4	0.35010 (16)	0.49101 (17)	0.28691 (14)	0.0467 (4)	
C5	0.29617 (18)	0.40323 (18)	0.22190 (14)	0.0539 (5)	
C6	0.37137 (19)	0.30638 (18)	0.20265 (15)	0.0569 (5)	
C7	0.50340 (19)	0.29551 (17)	0.24809 (14)	0.0520 (4)	
C8	0.55681 (15)	0.38361 (13)	0.31437 (12)	0.0401 (4)	
C9	0.7934 (11)	0.3110 (9)	0.3708 (9)	0.040 (2)	0.61 (2)
C10	0.7877 (9)	0.2168 (11)	0.4317 (7)	0.059 (2)	0.61 (2)
H10A	0.7200	0.2113	0.4686	0.070*	0.61 (2)
C11	0.8827 (10)	0.1295 (8)	0.4386 (7)	0.079 (3)	0.61 (2)
H11A	0.8793	0.0648	0.4802	0.094*	0.61 (2)
C12	0.9831 (7)	0.1383 (7)	0.3834 (8)	0.067 (5)	0.61 (2)
H12A	1.0474	0.0794	0.3891	0.081*	0.61 (2)
C13	0.9900 (11)	0.2300 (12)	0.3216 (9)	0.062 (3)	0.61 (2)
H13A	1.0569	0.2342	0.2839	0.074*	0.61 (2)
C14	0.8935 (12)	0.3191 (8)	0.3156 (10)	0.044 (2)	0.61 (2)
C9'	0.7814 (16)	0.3002 (12)	0.3785 (15)	0.041 (4)	0.39 (2)
C10'	0.7798 (10)	0.1928 (12)	0.4275 (10)	0.042 (3)	0.39 (2)
H10B	0.7110	0.1754	0.4611	0.050*	0.39 (2)
C11'	0.8812 (10)	0.1116 (7)	0.4262 (9)	0.062 (4)	0.39 (2)
H11B	0.8801	0.0397	0.4589	0.074*	0.39 (2)
C12'	0.9841 (11)	0.1377 (11)	0.3759 (12)	0.078 (9)	0.39 (2)
H12B	1.0519	0.0833	0.3751	0.094*	0.39 (2)
C13'	0.9857 (15)	0.2451 (15)	0.3270 (14)	0.053 (5)	0.39 (2)
H13B	1.0546	0.2625	0.2933	0.063*	0.39 (2)
C14'	0.8844 (19)	0.3263 (12)	0.3282 (16)	0.051 (5)	0.39 (2)
C15	0.53832 (16)	0.66877 (14)	0.44448 (12)	0.0427 (4)	
C16	0.64660 (19)	0.73974 (16)	0.50983 (16)	0.0537 (5)	
H5	0.2000 (19)	0.4086 (16)	0.1867 (14)	0.054 (5)*	
H4	0.305 (2)	0.5574 (17)	0.3006 (15)	0.062 (6)*	
H6	0.3308 (18)	0.2474 (17)	0.1547 (14)	0.058 (5)*	
H7	0.555 (2)	0.240 (2)	0.2376 (15)	0.072 (7)*	
H16A	0.6823 (19)	0.6949 (18)	0.5708 (16)	0.063 (6)*	
H16B	0.728 (2)	0.7566 (19)	0.4713 (15)	0.074 (6)*	

### Atomic displacement parameters (Å^2^)

**Table:**

	*U*^11^	*U*^22^	*U*^33^	*U*^12^	*U*^13^	*U*^23^
Br1	0.0676 (9)	0.0545 (7)	0.0882 (9)	0.0102 (3)	0.0310 (8)	0.0153 (4)
Br1'	0.113 (3)	0.0880 (14)	0.131 (2)	0.0047 (11)	0.0714 (17)	0.0209 (9)
Cl1	0.0333 (2)	0.0777 (3)	0.0813 (4)	0.0123 (2)	−0.0078 (2)	−0.0336 (3)
Cl2	0.1014 (4)	0.0418 (3)	0.0902 (4)	0.0102 (3)	0.0213 (3)	−0.0139 (2)
O1	0.0450 (7)	0.0610 (8)	0.0766 (9)	0.0230 (6)	0.0063 (6)	−0.0095 (7)
N1	0.0339 (7)	0.0424 (7)	0.0506 (8)	0.0114 (6)	0.0025 (6)	−0.0093 (6)
C1	0.0295 (7)	0.0446 (8)	0.0488 (10)	0.0049 (7)	0.0032 (7)	−0.0088 (7)
C2	0.0321 (7)	0.0402 (8)	0.0453 (9)	0.0058 (6)	0.0081 (7)	−0.0018 (7)
C3	0.0322 (7)	0.0397 (8)	0.0410 (9)	0.0013 (6)	0.0052 (7)	0.0043 (7)
C4	0.0318 (8)	0.0526 (10)	0.0545 (11)	0.0037 (8)	0.0056 (7)	0.0118 (9)
C5	0.0382 (9)	0.0636 (12)	0.0552 (12)	−0.0099 (8)	−0.0025 (8)	0.0127 (9)
C6	0.0576 (12)	0.0555 (11)	0.0526 (11)	−0.0156 (9)	−0.0020 (9)	0.0019 (9)
C7	0.0556 (11)	0.0442 (10)	0.0536 (11)	0.0014 (9)	0.0045 (9)	−0.0036 (8)
C8	0.0348 (8)	0.0414 (8)	0.0429 (9)	0.0026 (7)	0.0042 (7)	0.0009 (7)
C9	0.040 (4)	0.037 (3)	0.042 (4)	0.017 (3)	0.006 (3)	−0.001 (2)
C10	0.073 (4)	0.051 (4)	0.059 (4)	0.024 (3)	0.032 (3)	−0.001 (3)
C11	0.125 (7)	0.064 (3)	0.058 (3)	0.044 (4)	0.046 (4)	0.021 (3)
C12	0.067 (8)	0.072 (9)	0.062 (6)	0.050 (7)	0.011 (5)	0.010 (5)
C13	0.055 (5)	0.075 (6)	0.057 (5)	0.037 (4)	0.015 (4)	−0.010 (4)
C14	0.044 (4)	0.052 (4)	0.040 (3)	0.011 (3)	0.015 (3)	−0.013 (2)
C9'	0.030 (5)	0.054 (8)	0.040 (6)	0.003 (4)	0.008 (4)	−0.010 (5)
C10'	0.045 (4)	0.029 (4)	0.048 (5)	0.009 (3)	0.001 (3)	0.000 (3)
C11'	0.060 (6)	0.049 (4)	0.069 (6)	0.038 (4)	−0.008 (5)	−0.018 (4)
C12'	0.091 (15)	0.080 (16)	0.072 (11)	0.033 (11)	0.038 (9)	−0.018 (9)
C13'	0.055 (8)	0.051 (6)	0.060 (7)	0.005 (6)	0.030 (6)	−0.014 (4)
C14'	0.053 (8)	0.045 (5)	0.056 (9)	0.019 (5)	0.010 (5)	−0.005 (5)
C15	0.0419 (9)	0.0396 (8)	0.0474 (10)	0.0100 (7)	0.0105 (7)	0.0022 (7)
C16	0.0566 (11)	0.0387 (9)	0.0651 (13)	0.0053 (8)	0.0101 (10)	−0.0075 (9)

### Geometric parameters (Å, º)

**Table:**

Br1—C14	1.892 (5)	C9—C14	1.381 (7)
Br1'—C14'	1.883 (6)	C10—C11	1.379 (7)
Cl1—C1	1.7025 (15)	C10—H10A	0.9300
Cl2—C16	1.7703 (18)	C11—C12	1.385 (7)
O1—C15	1.2147 (18)	C11—H11A	0.9300
N1—C1	1.365 (2)	C12—C13	1.346 (7)
N1—C8	1.3939 (19)	C12—H12A	0.9300
N1—C9'	1.432 (6)	C13—C14	1.404 (7)
N1—C9	1.444 (4)	C13—H13A	0.9300
C1—C2	1.374 (2)	C9'—C10'	1.3900
C2—C3	1.452 (2)	C9'—C14'	1.3900
C2—C15	1.463 (2)	C10'—C11'	1.3900
C3—C8	1.401 (2)	C10'—H10B	0.9300
C3—C4	1.405 (2)	C11'—C12'	1.3900
C4—C5	1.373 (3)	C11'—H11B	0.9300
C4—H4	0.92 (2)	C12'—C13'	1.3900
C5—C6	1.395 (3)	C12'—H12B	0.9300
C5—H5	1.007 (19)	C13'—C14'	1.3900
C6—C7	1.375 (3)	C13'—H13B	0.9300
C6—H6	0.969 (19)	C15—C16	1.509 (2)
C7—C8	1.384 (2)	C16—H16A	0.98 (2)
C7—H7	0.85 (2)	C16—H16B	1.08 (2)
C9—C10	1.359 (7)		
			
C1—N1—C8	108.06 (12)	C12—C11—H11A	120.1
C1—N1—C9'	128.6 (9)	C13—C12—C11	121.6 (4)
C8—N1—C9'	122.6 (9)	C13—C12—H12A	119.2
C1—N1—C9	126.0 (6)	C11—C12—H12A	119.2
C8—N1—C9	125.9 (6)	C12—C13—C14	118.4 (5)
C9'—N1—C9	8.5 (10)	C12—C13—H13A	120.8
N1—C1—C2	111.46 (13)	C14—C13—H13A	120.8
N1—C1—Cl1	117.90 (11)	C9—C14—C13	120.3 (5)
C2—C1—Cl1	130.61 (13)	C9—C14—Br1	117.9 (7)
C1—C2—C3	105.22 (14)	C13—C14—Br1	121.7 (7)
C1—C2—C15	130.08 (15)	C10'—C9'—C14'	120.0
C3—C2—C15	124.70 (13)	C10'—C9'—N1	128.1 (11)
C8—C3—C4	117.94 (16)	C14'—C9'—N1	111.9 (11)
C8—C3—C2	107.47 (13)	C9'—C10'—C11'	120.0
C4—C3—C2	134.58 (16)	C9'—C10'—H10B	120.0
C5—C4—C3	118.80 (18)	C11'—C10'—H10B	120.0
C5—C4—H4	124.5 (13)	C12'—C11'—C10'	120.0
C3—C4—H4	116.7 (13)	C12'—C11'—H11B	120.0
C4—C5—C6	121.70 (17)	C10'—C11'—H11B	120.0
C4—C5—H5	119.5 (11)	C11'—C12'—C13'	120.0
C6—C5—H5	118.8 (11)	C11'—C12'—H12B	120.0
C7—C6—C5	121.01 (18)	C13'—C12'—H12B	120.0
C7—C6—H6	119.6 (11)	C12'—C13'—C14'	120.0
C5—C6—H6	119.4 (11)	C12'—C13'—H13B	120.0
C6—C7—C8	117.03 (18)	C14'—C13'—H13B	120.0
C6—C7—H7	125.0 (14)	C13'—C14'—C9'	120.0
C8—C7—H7	118.0 (14)	C13'—C14'—Br1'	114.9 (9)
C7—C8—N1	128.72 (15)	C9'—C14'—Br1'	123.5 (9)
C7—C8—C3	123.51 (15)	O1—C15—C2	120.30 (15)
N1—C8—C3	107.76 (13)	O1—C15—C16	121.46 (15)
C10—C9—C14	120.2 (4)	C2—C15—C16	118.24 (13)
C10—C9—N1	113.1 (9)	C15—C16—Cl2	112.50 (12)
C14—C9—N1	126.6 (9)	C15—C16—H16A	109.1 (11)
C9—C10—C11	119.7 (4)	Cl2—C16—H16A	110.2 (12)
C9—C10—H10A	120.1	C15—C16—H16B	111.0 (11)
C11—C10—H10A	120.1	Cl2—C16—H16B	106.4 (11)
C10—C11—C12	119.8 (4)	H16A—C16—H16B	107.5 (16)
C10—C11—H11A	120.1		
			
C8—N1—C1—C2	−0.4 (2)	C9'—N1—C9—C14	168 (10)
C9'—N1—C1—C2	−170.9 (8)	C14—C9—C10—C11	0.2 (7)
C9—N1—C1—C2	178.9 (6)	N1—C9—C10—C11	179.1 (11)
C8—N1—C1—Cl1	177.68 (12)	C9—C10—C11—C12	0.0 (9)
C9'—N1—C1—Cl1	7.2 (8)	C10—C11—C12—C13	−0.7 (9)
C9—N1—C1—Cl1	−3.0 (6)	C11—C12—C13—C14	1.2 (8)
N1—C1—C2—C3	1.0 (2)	C10—C9—C14—C13	0.3 (7)
Cl1—C1—C2—C3	−176.78 (15)	N1—C9—C14—C13	−178.4 (12)
N1—C1—C2—C15	−178.77 (16)	C10—C9—C14—Br1	−176.1 (10)
Cl1—C1—C2—C15	3.4 (3)	N1—C9—C14—Br1	5.2 (10)
C1—C2—C3—C8	−1.20 (19)	C12—C13—C14—C9	−1.0 (7)
C15—C2—C3—C8	178.58 (15)	C12—C13—C14—Br1	175.3 (11)
C1—C2—C3—C4	179.79 (19)	C1—N1—C9'—C10'	100.8 (14)
C15—C2—C3—C4	−0.4 (3)	C8—N1—C9'—C10'	−68.5 (16)
C8—C3—C4—C5	0.0 (2)	C9—N1—C9'—C10'	176 (11)
C2—C3—C4—C5	178.90 (19)	C1—N1—C9'—C14'	−80.0 (9)
C3—C4—C5—C6	−0.2 (3)	C8—N1—C9'—C14'	110.7 (8)
C4—C5—C6—C7	−0.3 (3)	C9—N1—C9'—C14'	−5 (9)
C5—C6—C7—C8	1.0 (3)	C14'—C9'—C10'—C11'	0.0
C6—C7—C8—N1	179.49 (17)	N1—C9'—C10'—C11'	179.2 (19)
C6—C7—C8—C3	−1.2 (3)	C9'—C10'—C11'—C12'	0.0
C1—N1—C8—C7	179.00 (18)	C10'—C11'—C12'—C13'	0.0
C9'—N1—C8—C7	−9.8 (8)	C11'—C12'—C13'—C14'	0.0
C9—N1—C8—C7	−0.3 (6)	C12'—C13'—C14'—C9'	0.0
C1—N1—C8—C3	−0.38 (18)	C12'—C13'—C14'—Br1'	−166.3 (16)
C9'—N1—C8—C3	170.8 (8)	C10'—C9'—C14'—C13'	0.0
C9—N1—C8—C3	−179.7 (6)	N1—C9'—C14'—C13'	−179.3 (16)
C4—C3—C8—C7	0.8 (3)	C10'—C9'—C14'—Br1'	165.0 (17)
C2—C3—C8—C7	−178.44 (16)	N1—C9'—C14'—Br1'	−14.2 (13)
C4—C3—C8—N1	−179.82 (15)	C1—C2—C15—O1	−174.92 (18)
C2—C3—C8—N1	0.98 (18)	C3—C2—C15—O1	5.4 (3)
C1—N1—C9—C10	99.5 (8)	C1—C2—C15—C16	5.1 (3)
C8—N1—C9—C10	−81.3 (8)	C3—C2—C15—C16	−174.67 (17)
C9'—N1—C9—C10	−11 (9)	O1—C15—C16—Cl2	−4.9 (2)
C1—N1—C9—C14	−81.7 (8)	C2—C15—C16—Cl2	175.12 (13)
C8—N1—C9—C14	97.4 (9)		

### Hydrogen-bond geometry (Å, º)

**Table:**

*D*—H···*A*	*D*—H	H···*A*	*D*···*A*	*D*—H···*A*
C4—H4···O1	0.92 (2)	2.502 (19)	3.053 (2)	118.5 (15)
C16—H16*A*···Cl1	0.98 (2)	2.759 (19)	3.1275 (18)	103.0 (13)
C10—H10*A*···O1^i^	0.93	2.45	3.302 (7)	152

Symmetry code: (i) −*x*+1, −*y*+1, −*z*+1.
